# Separation of transglutaminase by thermo-responsive affinity precipitation using l-thyroxin as ligand

**DOI:** 10.1186/s40064-016-1680-0

**Published:** 2016-01-15

**Authors:** Sipeng Li, Zhaoyang Ding, Xuejun Cao

**Affiliations:** State Key Laboratory of Bioreactor Engineering, Department of Bioengineering, East China University of Science and Technology, Shanghai, 200237 China

**Keywords:** Affinity precipitation, Transglutaminase, Thermo-responsive polymer, l-Thyroxin

## Abstract

Transglutaminase (TGase) is widely used in the food industry. In this study, TGase was purified by affinity precipitation using l-thyroxin, coupled to a thermo-responsive polymer (P_NBN_), as an affinity ligand. The lower critical solution temperature and recovery of the affinity polymer were 31.0 °C and 99.6 %, respectively. The optimal adsorption condition was 0.02 mol/L phosphate buffer (pH 5.0). The recoveries 99.01 % (protein) and 98.85 % (activity) were obtained by 0.2 mol/L Gly–NaOH buffer (pH 10.0) as the elution agent. Circular dichroism spectroscopy and FortéBio Octet system were used to explore the interactions between l-thyroxin and TGase. The results show that l-thyroxin is suitable for affinity precipitation of TGase. The purity of the final product was verified using sodium dodecyl sulfate polyacrylamide gel electrophoresis.

## Background

Transglutaminase (TGase; EC 2.3.2.13) is an enzyme that can catalyze the acyl-transfer reaction from the γ-carboxamide group of a glutamine residue to the ε-amino group of a lysine residue (Grossowicz et al. 1950; Folk 1980). The cysteine at the active site of TGase can generate covalent cross-links with the glutamyl residue, which acts as an acyl donor, resulting in ε-(*γ*-Glu)-Lys bonds among proteins or peptides (Chica et al. 2004). When water molecules act as the acyl receptor, the deamidation of the glutamine and formation of a glutamine acid occur (Pardin et al. 2009). This enzyme therefore has a wide range of applications in food, drugs, immune studies, and metabolism (Binsi and Shamasundar 2012).

The forms and sources of TGase are diverse, and it can be obtained from plants, mammals, microorganisms, fish, and some marine organisms. Based on the origin and properties of the enzyme, TGases can be divided into tissue TGase and microbial TGase (Wilhelm et al. 1996; Worratao and Yongsawatdigul 2005). TGase from microorganism has been widely used in the food industry since the early 1980s (Zhao et al. 2010). Proteins play an important role in the nutritional values and composition of most foods, and TGase can cross-link many important food proteins such as casein and soy proteins. These modifications by TGase affect the solubility, gelation, emulsification, and other properties of the proteins. To acquire the high-purity and high-activity TGase with food industry standard is still a challenge for bioseparation technology (Luciano and Arntfield 2012).

The applications of commercial TGase products require suitable purification methods. There are various methods for TGase purification, such as ammonium sulfate precipitation, organic solvent precipitation, size-exclusion chromatography, ion-exchange chromatography, and affinity chromatography (Hemung and Yongsawatdigul 2008). Precipitations using ammonium sulfate or organic solvents have low selectivity. Size-exclusion, ion exchange, and affinity chromatography methods are complicated, inefficient, and expensive for large-scale production. This article describes an affinity precipitation method for TGase purification.

Affinity precipitation is a useful technique. The target protein could be precipitated from the crude culture by reversible soluble–insoluble polymer coupled with an affinity ligand (Ding et al. 2014a; Arnold and Chen 2014; Wu et al. 2006). The affinity polymer can be recycled and the affinity ligand on the polymer has high selectivity, so affinity precipitation has already been studied for some bioproducts (Capito et al. 2013; Gagnon et al. 2011; Janoschek et al. 2014; Zhou et al. 2010). A thermoresponsive polymer, 1-vinylimidazole *N*-isopropylacrylamide, with Cu^2+^ or Ni^2+^ as the affinity ligand were synthesized to purify His-tag proteins (Mattiasson et al. 2007). The haptoglobin was used as an affinity ligand connected to a thermoresponsive polymer, poly(*N*-isopropylacrylamide), and the results showed that this method is simpler and has higher purification efficiency than others (Stocker-Majd et al. 2008). A pH-responsive polymer with Cibacron Blue F3GA was applied as the affinity polymer, to purify cellulase. The final purity and activity of the cellulase were 84.4 and 99.8 %, respectively (Ding and Cao 2013). The PNVCL-*co*-MAA-Cu^2+^ was used to precipitate bovine serum albumen (BSA) and the maximum amount of eluted BSA was 37.3 mg/g polymer (Ling et al. 2012). l-thyroxin has first been used as affinity ligand in our group because of its high selectivity and safety. In this study, l-thyroxin was immobilized to a polymer as the affinity polymer for TGase purification. Affinity process and molecular mechanism have been explored. The study is hopeful to improve present purification process of TGase and to understand molecular mechanism of affinity precipitation.

## Methods

### Materials

*N*-methylolacrylamide (NMAM), *N*-isopropylacrylamide (NIPA), azobisisobutyronitrile (AIBN), and butyl acrylate (BA) were purchased from the Sinopharm Chemical Reagent Co., Ltd. (Shanghai, China). l-Thyroxin, pure TGase, l-glutaminehydroxylamine, and *N*-carboxybenzoyl-l-glutaminyl-glycine (*N*-CBZ-Gln-Gly) were purchased from Sigma (St. Louis, MO, USA). All other chemicals were of analytical reagent grade.

### Interactions between l-thyroxin and TGase

The interactions between l-thyroxin and TGase were investigated using circular dichroism (CD) spectroscopy and FortéBio Octet system (FortéBio Inc., USA) to detect micro-changes in protein properties and structures when they were combined with other molecules.

### CD spectroscopy

Different asymmetric molecules absorb different amounts of circularly polarized light. The CD spectrum shows the relationship between the absorbed amount and wavelength of the light (Brkljaca et al. 2012). CD spectrum was used to compare the changes in the secondary structure of TGase in the presence of different concentrations of l-thyroxin.

### FortéBio Octet system

Biolayer interferometry is a key part of the FortéBio Octet system and the system is used to perform label-free analysis of protein-binding interactions in real time, and is a non-flow dip-and-read system (Li et al. 2011). The analyte is placed in the microplate wells, and ligand-coated sensor tips are immersed in the wells. The system detects the interactions in each well. The affinity polymer were biotinylated by mixing with equivalent amounts of biotin for 30.0 min, then using a PD-10 desalting column (GE Healthcare, USA) to wash out unreacted biotin. The sensors (Super Streptavidin, SSA) were prewetted in dialysis buffer for 15.0 min, held in the biotinylated polymer solution for 15.0 min, and quenched in 10.0 μmol/L biotin for 1.0 min. The unloaded biotinylated polymer was used as controls to calibrate the baseline drift. All these polymers were reacted with l-thyroxin solution, which was prepared in a series of dilutions (0.5, 1.0, 1.5, 2.0, and 2.5 μmol/L) at room temperature. All tests were performed in triplicate.

### Affinity polymer preparation

#### Polymer synthesis

P_NBN_ is a reversible soluble–insoluble polymer that is responsive to temperature changes (Ding and Cao 2013; Shen and Cao 2007). This property comes from the NIPA monomer. NIPA (7.91 g), BA (0.50 mL), and 0.50 g NMAM (0.50 g) were dissolved in ethanol (100 mL). O_2_ was removed using N_2_, and AIBN (0.05 g) was added as the initiator. The reaction was performed in a constant-temperature bath at 60 °C for 24 h. After the reaction completed, the polymer was obtained by vacuum distillation. The precipitate was dissolved in acetone and extracted with *n*-hexane. The white product was obtained after drying in a vacuum.

#### Affinity ligand immobilization

**Fig. 1:**
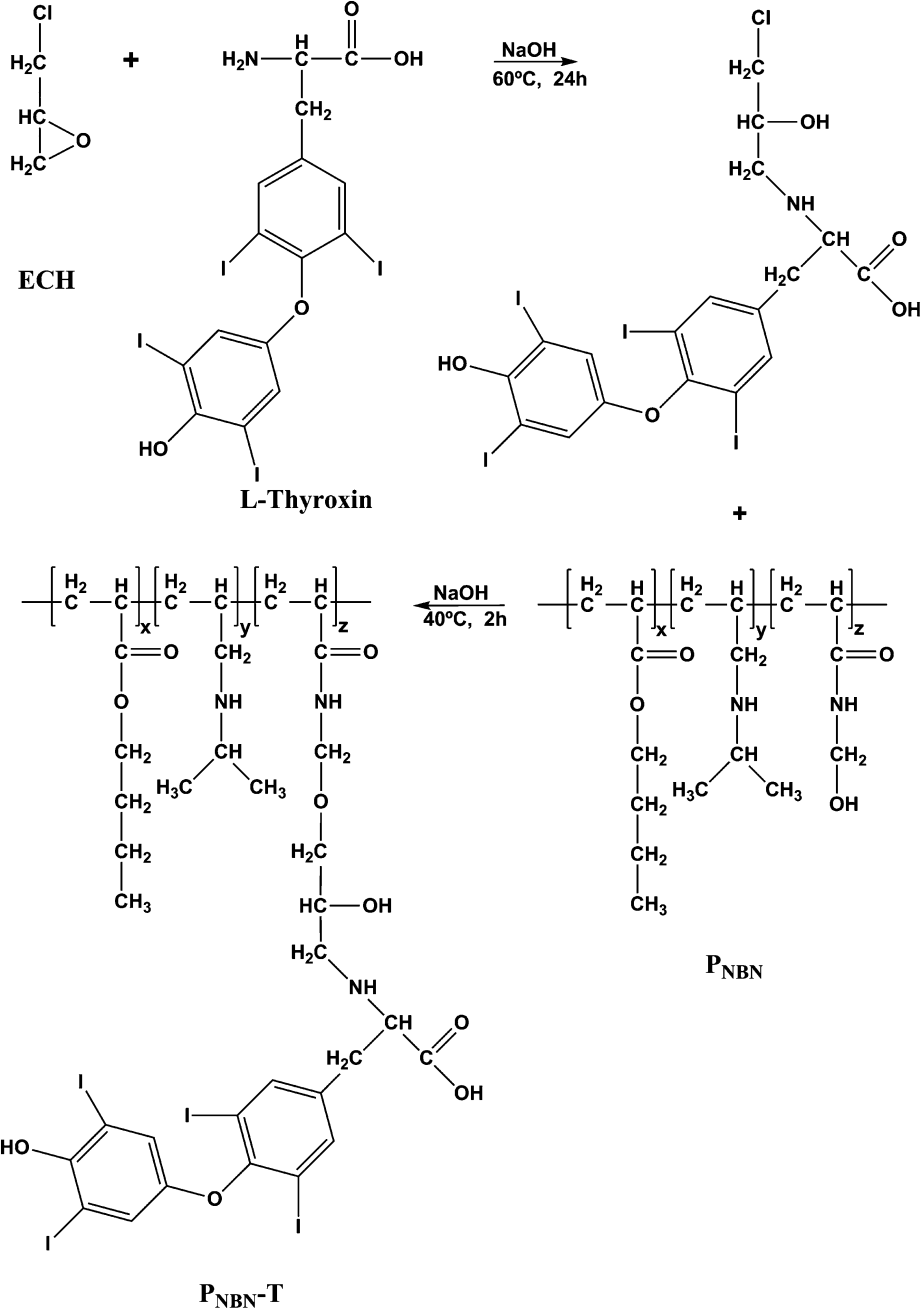
Immobilization of l-thyroxin to P_NBN_. l-Thyroxin was firstly reacted with the epichlorohydrin (ECH) in the NaOH solution (1 mol/L) at 60 °C for 24 h. Then the compounds were combined with P_NBN_ through the hydroxyl groups at the conditions of 1 mol/L NaOH, 40 °C, 2 h

Epichlorohydrin (ECH) was introduced to connect the affinity ligand and the polymer (Fig. 1). The polymer, P_NBN_ (1 %, w/v), was dissolved in a 1 mol/L NaOH solution and then 0.5 % (v/v) ECH was added. The reaction was performed in a constant-temperature bath at 60 °C for 24 h. Different amounts of l-thyroxin were reacted with the polymer–ECH at 40 °C for 2 h, and the P_NBN_–thyroxin was precipitated by changing the temperature. The supernatant was collected and the ligand density of the P_NBN_–thyroxin was determined using the mass balance calculation.

#### Measurement of l-thyroxin by HPLC

A modified method with HPLC system (Shimadzu, Japan) was performed. An analytical reversed-phase column (ZORBAX C18, 4.6 × 150 mm) was used at the flow rate (1.0 mL/min). All the measurements were performed at room temperature and the samples were detected by UV at 212 nm. The optimal mobile phase used was a mixture of solvent A: 0.1 % phosphoric acid in methanol and solvent B: 0.1 % phosphoric acid in water.

### Determination of TGase activity

The TGase activity was determined based on hydroxamate formation with the substrate, *N*-CBZ-Gln-Gly (Grossowicz et al. 1950). Solution A consisted of hydroxylamine hydrochloride (0.3475 g), reduced glutathione (0.1537 g), and *N*-CBZ-Gln-Gly (0.5060 g) dissolved in 0.2 mol/L Tris–HCl buffer (pH 6.0, 50 mL). The final pH of solution A was adjusted to 6.0. Solution B consisted of equal volumes of 5 % FeCl_3_·6H_2_O solution (dissolved in 0.1 mol/L HCl), 3 mol/L HCl, and 12 % trichloroacetic acid.

A standard curve was obtained as follows. TGase solutions of different concentrations (0.2 mL) were mixed with solution A (0.8 mL) and kept in water bath (37 °C) for 10 min. Then solution B (1.0 mL) was added to terminate the reaction. After centrifugation for 5 min at 5000 rpm, the absorbance of the supernatant at 525 nm was measured. The TGase activities of the samples were determined using the method described above.

### Adsorption of TGase

P_NBN_–thyroxin was dissolved in water at a 4 % (w/v) concentration, and 0.4 mg/mL TGase was prepared with 0.2 mol/L phosphate buffer (PB, pH 7.0). Equal volumes of these two solutions were mixed, and the mixture was shaken at 100 rpm for 2 h at room temperature. A precipitate was obtained by adjusting the temperature of the resultant solution and centrifugation. The supernatant was used for determination of the adsorption ratio from the material balance. During these procedures, parameters such as reaction time, pH, ionic strength, and ligand density which affected the adsorption capacity, were examined. Adsorption isotherms were determined (Pavan et al. 2014).

### Elution of TGase

After precipitation, TGase was eluted from the affinity polymer by breaking the bonding between TGase and l-thyroxin. Different elution agents were used to test elution effect activity of TGase. The precipitated mixture (1.0 g) was dissolved in the elution agents (5.0 mL) and shaken at 200 rpm for 2 h. Then P_NBN_–thyroxin was precipitated by changing the temperature to above the lower critical solution temperature (LCST), and the supernatant was used for determination of the TGase activity. The P_NBN_–thyroxin was recycled for the next affinity precipitation.

### Affinity precipitation of TGase from crude TGase

Affinity precipitation of TGase from crude TGase was carried out in the same process as above. The optimal adsorption and desorption conditions obtained above were applied in this experiment. The eluted TGase was assayed using SDS-PAGE (Laemmli 1970). 15.0 % acrylamide gels were used in the experiments, and the final gels were stained with 0.25 % Coomassie Brilliant Blue R-250. Crude TGase and pure TGase were used as controls.

### Recycle of affinity polymer

After desorption experiments of the TGase, the affinity polymer was recovered and regenerated with the suitable eluent. The regenerated polymer was reused in the next cycle of purification experiments.

## Results and discussion

### CD spectrum

**Fig. 2:**
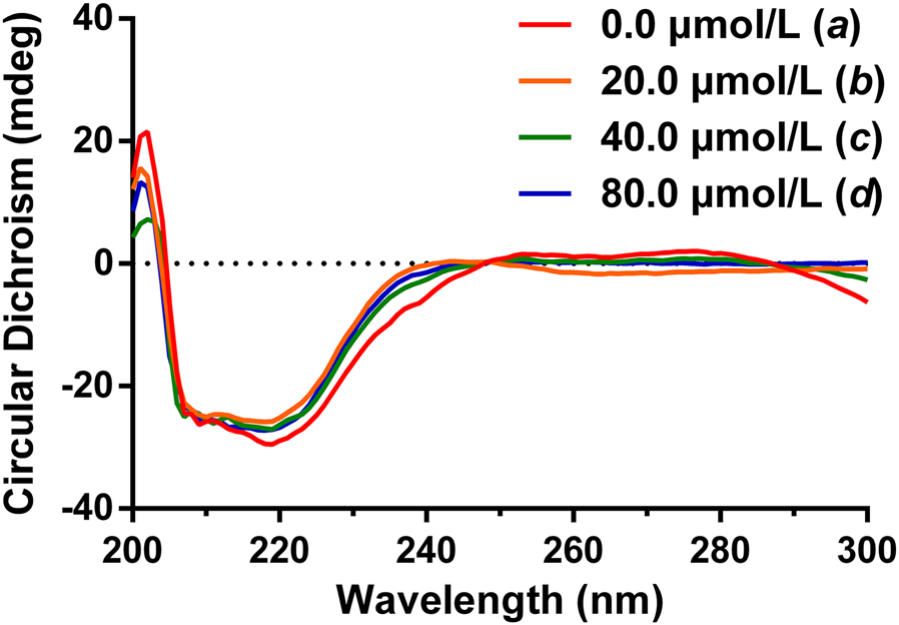
Interaction CD spectra of TG and l-thyroxin system. Spectra were obtained in 1.0 μmol/L PB (pH 7.0, room temperature). TGase concentration was kept at 10.0 μmol/L. In the TGase and l-thyroxin systems, the l-thyroxin concentrations were 0.0 μmol/L (*a*), 20.0 μmol/L (*b*), 40.0 μmol/L (*c*), and 80.0 μmol/L (*d*)

Figure 2 shows the CD spectra of TGase and l-thyroxin. The CD spectrum of TGase had a negative band in the UV region near 220 nm, which is characteristic of an α-helical structure, and the α-helix ratio in the secondary structure of TGase decreased from 31.98 % (*a*) to 28.26 % (*b*), 25.45 % (*c*) or 21.08 % (*d*) as in Fig. 2 by adding different concentrations of l-thyroxin. These results indicate that the ligand underwent interactions with TGase, and that increasing the amount of l-thyroxin caused greater changes in the secondary structure of TGase. This demonstrated that there was a specific affinity interaction between l-thyroxin and TGase.

### FortéBio Octet system

**Fig. 3:**
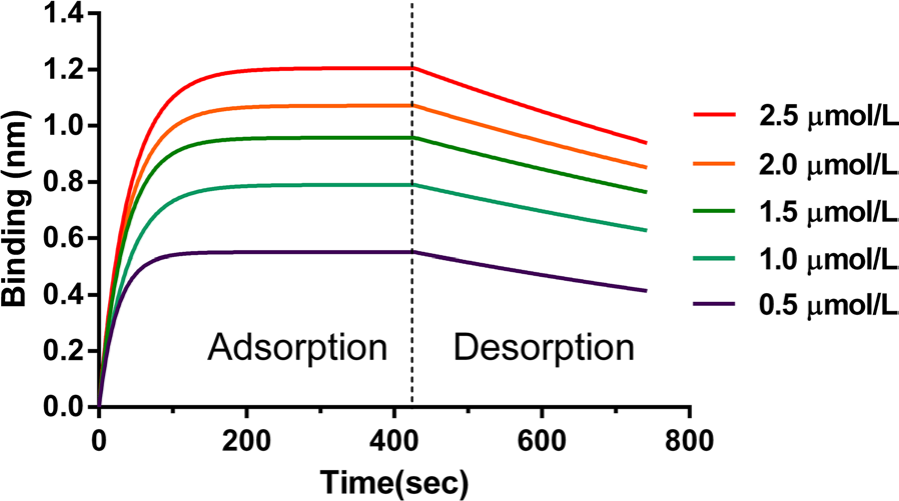
The results of FortéBio Octet assay. *Vertical* and *horizontal axes* represent light shift distance (nm) for different concentrations of TGase in solution and adsorption/desorption time (s)

In Fig. 3, the vertical axes represent the adsorbed amount. The results show that adsorption equilibrium time was affected by the initial concentration of l-thyroxin. And with the increase of it, the absorbed amounts grew sharply. According to these tendencies, the dissociation constant (*K*_*d*_) for this system could be calculated as 18.34 ± 2.15 μmol/L. This showed that l-thyroxin had sufficient TGase affinity strength, and was therefore a suitable ligand for affinity precipitation of TGase.

### Affinity precipitation of TGase

**Fig. 4:**
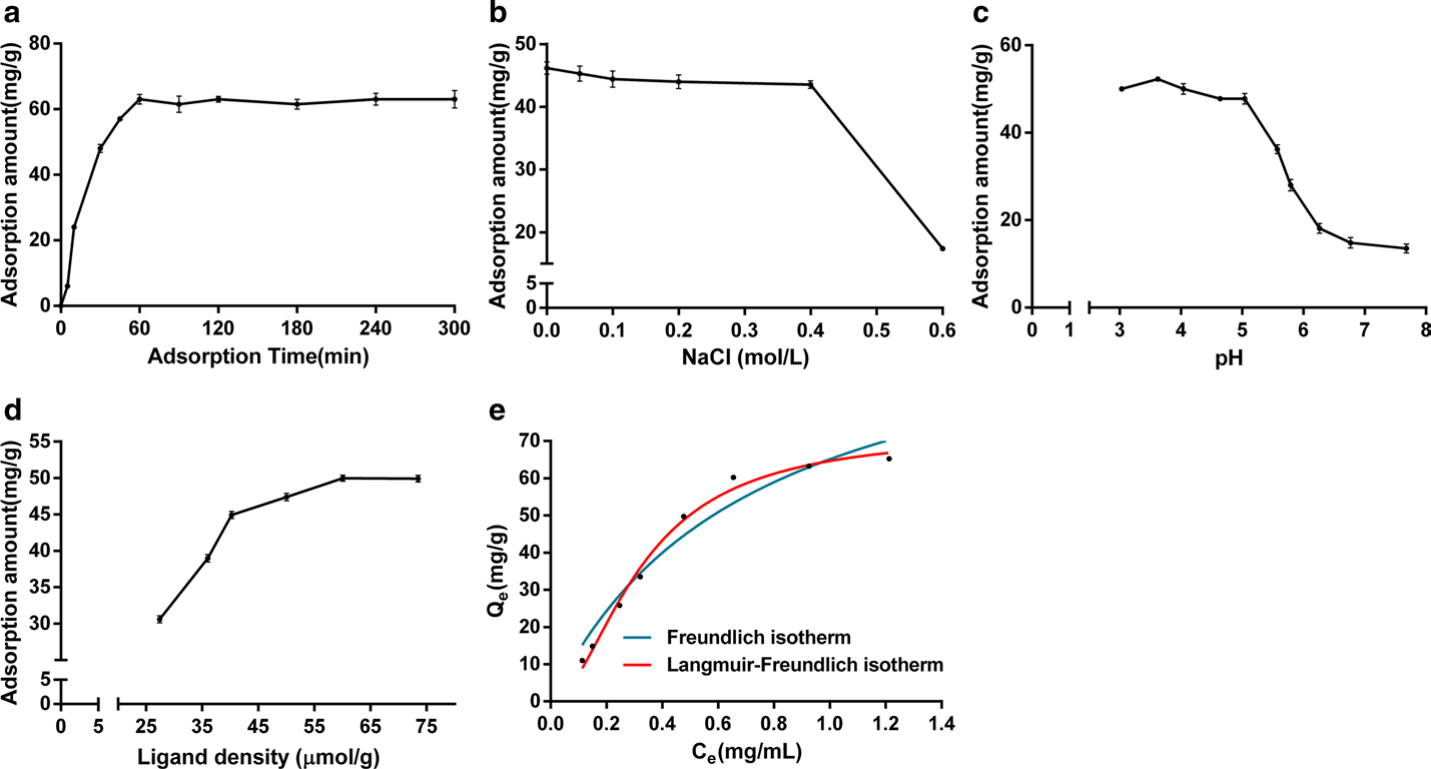
Affinity precipitation of TGase. Initial concentration of TGase 2.0 mg/mL, 20.0 °C, pH 7.0, affinity ligand density 59.5 μmol/g, and 0.1 g polymer. **a** Effect of reaction time on adsorption on P_NBN_–thyroxin. **b** Effect of pH of P_NBN_–thyroxin on TGase binding. **c** Effect of ionic strength of P_NBN_–thyroxin on TGase binding. **d** Effect of ligand density on P_NBN_–thyroxin on TGase binding. **e** Adsorption isotherms of TGase binding to affinity polymer. Fitting plot of adsorption isotherm by Freundlich isotherm (*blue line*) and Langmuir–Freundlich isotherm (*red line*). *Q*_*e*_ is the amount of TGase adsorbed per unit of the affinity polymer; *C*_*e*_ is the equilibrium concentration of the TGase in the solution

Figure 4a shows that the adsorbed TGase amount increased rapidly at the beginning, while adsorption equilibrium was reached in 60 min and kept stable. The process was influenced by the concentration of TGase in the solution and the adsorption capacity of P_NBN_–thyroxin.

The effect of pH on adsorption is illustrated in Fig. 4b. The optimal adsorption was achieved at pH 3.5. The adsorbed TGase amount decreased with pH increasing. This is because the net charge on the TGase surface approached 0 as the pH approached the isoelectric point (9.0) of TGase. This reduced binding between l-thyroxin and TGase to result in lower adsorbed TGase amount. To optimize the final TGase activity achieved, the pH should be controlled at 5.0–8.0. To ensure the TGase stability, pH 5.0 was selected.

The adsorbed amount and activity of TGase were both considered in this assay, because a high ionic strength might affect determination of the TGase activity. As shown in Fig. 4c, high NaCl concentration decreased the adsorbed amount and activity simultaneously. High ionic strength would destroy the hydrophilicity of the P_NBN_–thyroxin, resulting in polymer precipitation, and weakening the binding between ligand and TGase to reduce adsorbed amount. A high ionic strength could also cause changes of the enzyme conformation. As a result, 0 mg/mL NaCl was chosen as the final selection.

Figure 4d shows the effect of ligand density on P_NBN_–thyroxin on the adsorbed TGase amount. When the ligand density was below 50.0 μmol/g, the adsorbed TGase amount increased with increasing of ligand density. While ligand density was above 50.0 μmol/g, adsorption amount approached to stable. At a low ligand density, the amount of TGase bound to the polymer would increase with ligand density. However, at a high ligand density, more ligands on the polymer would increase steric hindrance, decreasing the possibility of TGase attaching to the ligand. Ligand density at 59.5 μmol/g was chosen for affinity precipitation of TGase.

In consideration of the characters of the affinity polymer and target protein, the Freundlich isotherm and Langmuir–Freundlich (LF) isotherm equations which were used to describe heterogeneous adsorption systems were selected to correlate the experimental data of adsorption isotherm of affinity precipitation (Dhawan et al. 2015).

The Freundlich isotherm relates the adsorbed concentration as the power function of free solute concentration. One limitation of the Freundlich model is that the amount of adsorbed solute increases indefinitely with the free solute concentration. This empirical equation takes the form (Arıca and Bayramoğlu 2005; Arıca et al. 2004).

1 $$Q_{e} = K_{F} C_{e}^{{b_{F} }}$$

where *b*_*F*_ is the Freundlich constant, *K*_*F*_ is the adsorption coefficient, *Q*_*e*_ (mg/g) is the amount of TGase adsorbed per unit of the affinity polymer, and *C*_*e*_ (mg/mL) is the free concentration of the TGase in the solution. This expression is characterized by the Freundlich constant, *b*_*F*_, and so the Freundlich isotherm may be used to describe heterogeneous systems.

For LF isotherm, it is also used to describe the adsorption from dilute solutions on heterogeneous surfaces. It has an advantage that it can estimate the value of maximum adsorption capacity. It can be expressed as followed (Umpleby et al. 2001; Umpleby II et al. 2001):

2 $$Q_{e} = Q_{m} aC_{e}^{m} /(1 + aC_{e}^{m} )$$

where *Q*_*m*_ (mg/g) is the maximum adsorption capacity, *a* is related to association constant (*K*_0_) via *K*_0_ = *a*^1/*m*^, *m* is heterogeneity parameter, *Q*_*e*_ and *C*_*e*_ is the same as above equation.

Figure 4e shows the fitting plot of *Q*_*e*_ versus *C*_*e*_ using two models above, and Table 1 presents the fitting parameters. As shown in the Table 1, the value of R^2^ of LF isotherm was 0.9970, which is better than the R^2^ (0.9820) of Freundlich isotherm, which meant LF equation had better consistency with the experimental data.

**Table 1:** Fitting parameters of Freundlich isotherm and Langmuir–Freundlich isotherm

Freundlich isotherm			Langmuir–Freundlich isotherm			
*K*_*F*_ (mg/mL)	*b*_*F*_	R^2^	*Q*_*m*_ (mg/g)	*a* (mg/mL)	*m*	R^2^
71.89	0.7153	0.9820	123.2	1.419	1.207	0.9970

As for Freundlich constant in Table 1, *b*_*F*_ (0.7153) was less than 1, because the surface of affinity polymer was heterogeneous, and the TGase concentration on the polymer would increase as long as there was an increase in the initial TGase concentration. Meanwhile, for LF isotherm, *Q*_*m*_ was 123.2 mg/g and *K*_0_ was 1.336. The fact that *m* value was not equal to 1 implied that the binding sites of affinity polymer were heterogeneous which identified with Freundlich isotherm. According to *K*_0_, the dissociation constant (*K*_*d*_) was 0.7483 mg/mL, via *K*_*d*_ = 1/*K*_0_. As the molecular weight of TGase is 38,000 Da, the value of *Q*_*m*_ and *K*_*d*_ could be converted to 3.242 μmol/g and 19.69 μmol/L. In comparison with the results of Octet system, the value was closed to each other within the accepted error range. LF isotherm equation was much more suitable for the simulation of this affinity precipitation isotherm.

### Elution

Ten elution conditions were used to determine the optimal elution condition, based on the properties of l-thyroxin and TGase. The data in Table 2 show that pH 10.0 Gly–NaOH buffer obtained 99.01 % protein recovery and 98.05 % activity recovery, respectively. 0.02 mol/L phosphate buffer (pH 7.0) with 0.5 mol/L urea obtained 99.12 % protein recovery, while TGase activity recovery was only 80.89 %. None of other groups gave sufficient high TGase activity recovery, so Gly–NaOH (pH 10.0) was chosen as the elution agent.

**Table 2:** Different elution agents and results

Group	Elution condition	Elution recovery of protein (%)	Elution recovery of activity (%)
1	0.5 mol/L NaSCN	56.30	54.01
2	1.0 mol/L NaSCN	84.01	85.10
3	0.1 mol/L EDTA	91.09	90.02
4	1 mol/L KSCN	85.79	79.34
5	pH7.0 PB + 0.5 mol/L Urea	99.12	80.89
6	pH7.0 PB +1.0 % Triton 100	17.85	10.98
7	pH7.0 PB + 20.0 % Glycol	89.96	85.09
8	pH8.0 Gly–NaOH	36.22	40.87
9	pH9.0 Gly–NaOH	56.50	61.40
10	pH10.0 Gly–NaOH	99.01	95.85

The traditional separation methods were usually applied for microbial TGase, such as ammonium sulfate precipitation, organic solvent precipitation, size-exclusion chromatography, ion-exchange chromatography and so on. A number of components existed in the fermentation broth resulted in the complicated process for separation of TGase. The group of Shi acquired the purified TGase through alcohol precipitation, ammonium sulphate precipitation, and ultrafiltration gel chromatography. The final purification factor was 10.42 and activity recovery was 27.3 % (Shi et al. 2011). A simple purification process was applied by Macedo et al. The process includes two successive chromatography processes with Sephadex G-75 columns, with 48 and 17 % recovery, respectively (Macedo et al. 2011).

### SDS-page

**Fig. 5:**
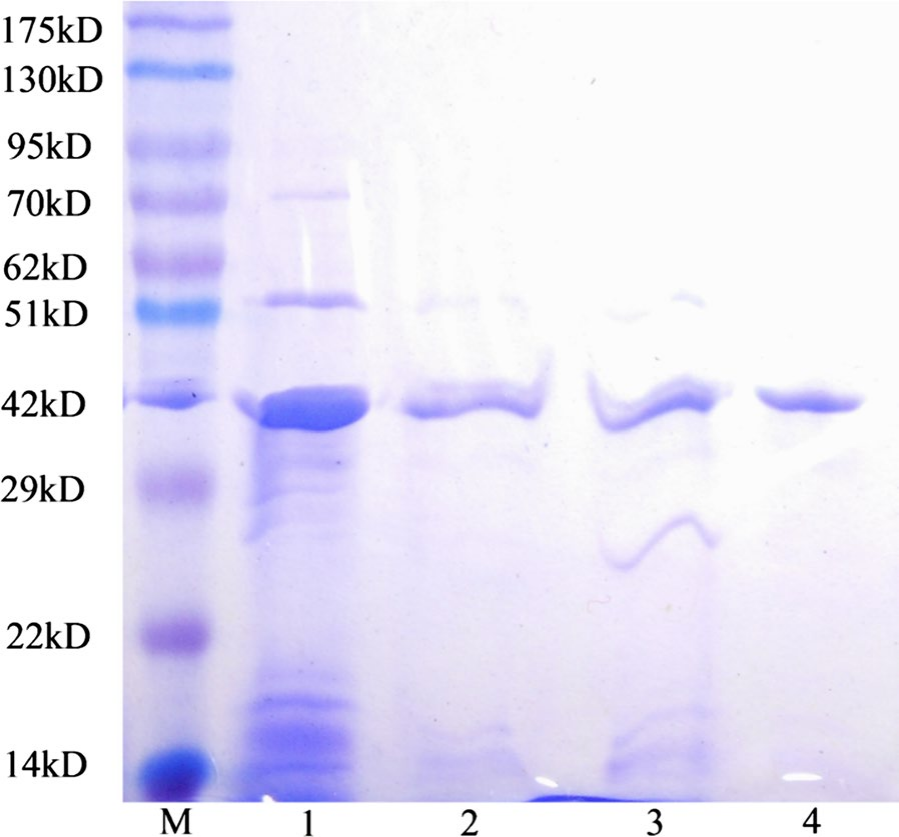
Purification of TGase by affinity precipitation. *M* marker, *1* crude TGase, *2* eluted with pH 10.0 PB, *3* eluted with pH 7.0 PB + 0.5 M urea, and *4* pure TGase

Crude TGase, pure TGase, and TGase purified by affinity precipitation were all analyzed using SDS-PAGE; and the results are shown in Fig. 5. Lanes 1 and 4 represent crude TGase and pure TGase, respectively. TGase eluted by Gly–NaOH (pH 10.0) is in lane 2, and lane 3 represents TGase eluted by PB (pH 7.0) containing 0.5 mol/L urea. For all four lanes, the TGase molecular weight was about 38 kD. The lane 2 of TGase purified by affinity precipitation show single band, while it seems that lane 3 show impurity bands. The results of lane 3 was caused by strong desorption and denaturation of urea for proteins which had other bands of different molecular weight. Pure TGase was obtained using affinity precipitation when Gly–NaOH (pH 10.0) was used as elution agent.

Rickert and the group used a two-step affinity purification method to achieve TGase of electrophoretic purity (Rickert et al. 2015). Compared with these methods mentioned above all, affinity precipitation is simpler and obtain higher yield and purity. In consideration of large-scale production for TGase, affinity precipitation is also a better choice.

### Recycle of P_NBN_–thyroxin

P_NBN_ was synthesized as described in the method. The LCST is 31.0 °C, and its recovery still reached 96.0 % at the five cycles with relation to initial amount, respectively. This shows that P_NBN_ was stable after repeated dissolution and precipitation. After l-thyroxin was attached to the polymer, the LCST is 31.6 °C, with 0.6 °C rising. Recovery of P_NBN_–thyroxin was 99.6 % at the five cycles with relation to initial amount. Theoretically, the affinity polymer could be recycled more than 60 times if recovery is over 95 % every recycle. This new affinity polymer can reduce the cost in a large extent, and show great potential application in industry.

Various kinds of proteins have different character for environmental stimulus. Aiming at the applicability of affinity precipitation, we have prepared diverse affinity precipitation systems with smart polymers (pH-responsive or thermos-responsive) to purify different proteins. Specific affinity ligands were also selected based on the interaction between the target proteins and ligands (Ding et al. 2014b; Zhou et al. 2010; Shen and Cao 2007).

## Conclusion

In this study, a thermo-responsive polymer P_NBN_ was synthesized and then ligand l-thyroxin was coupled to the polymer. The LCST of the affinity polymer closed to room temperature was useful for reducing energy consumption and retaining high activity of the enzyme. Furthermore, the high recycling ratio of the affinity polymer could ensure its reusability. Optimal adsorptions were achieved at a suitable adsorption condition of pH = 5.0 and 0 mg/mL NaCl. And Gly–NaOH (pH 10.0) was used as the best elution condition in which 99.01 % protein recovery and 98.05 % activity recovery was obtained, respectively. The SDS-PAGE showed the molecular weight of main band was corresponding to that of the native TGase (38 kD). The results reported here indicated that TGase could be efficiently purified using affinity precipitation.
